# Evolution of fairness in the divide-a-lottery game

**DOI:** 10.1038/s41598-023-34131-w

**Published:** 2023-04-29

**Authors:** Jeong-Yoo Kim, Kyu-Min Lee

**Affiliations:** 1 Department of Economics, Kyung Hee University, 1 Hoegi-dong, Dongdaemun-gu, Seoul, 137-701 Republic of Korea; 2 College of Business, Korea Advanced Institute of Science and Technology, Seoul, 02455 Republic of Korea

**Keywords:** Evolution, Cultural evolution, Evolutionary theory, Social evolution

## Abstract

In this paper, we show that fairness can evolve in the divide-a-lottery game which is more general than the divide-a-dollar game by using an indirect evolutionary approach. In the divide-a-lottery game, the size of a pie is uncertain. Two players sequentially bid for a share and they get their bid if the allocation based on the bids turns out to be feasible and otherwise neither gets anything. In this game, rational players over-compete for a higher share, resulting in a high probability of failure in agreement, whereas fair players who dislike the disparity between shares lower their bids thereby reducing the failure probability and thus increasing the expected payoff. As a result, fairness strictly dominates rationality. This is the mechanism through which fairness evolves. However, this result is not robust against even a slight uncertainty about the opponent’s type. Surprisingly, we show a contrasted simulation result that only rational players who are strictly dominated by fair players survive evolutionarily for most of the parameter values if players have even a slight chance of not knowing the opponent’s type. Our simulation results in a local interaction model in which players only know the type of closer neighbors capture both insights and demonstrate that moderate proportions of both types coexist evolutionarily over time, and that the population average fitness of this polymorphic population is higher than monomorphic population consisting only of fair types or rational types.

## Introduction

Although most economists and game theorists assume that material self-interest is the sole motivation of people, there is overwhelming counter-evidence gathered by psychologists and experimental economists. This evidence indicates that a substantial percentage of human beings are strongly motivated by other-regarding preferences including fairness, altruism etc. (For recent theoretical developments in evolution of prosocial cooperative behavior in various situations, e.g., in heterogeneous network structures, directional networks, and multilayer interactions, see McAvoy et al.^1^, Su et al.^2,3^). Considering that the selfish behavior, by definition, maximizes the individual’s utility or fitness and thus only *homo economicus* appears to be able to survive in the long run, it is rather puzzling that fair behavior survives in the long run in an evolutionary environment. The evidence of fairness is, however, well documented. For example, in the ultimatum game, a robust result across hundreds of experiments is that the vast majority of the offers are between 40 and 50 percent of the available surplus (see, for example, Güth et al.^4^, Camerer and Thaler^5^, Roth^6^, Camerer^7^).

In this paper, we show that fairness can evolve in the divide-a-lottery game which is more general than the divide-a-dollar game by using an indirect evolutionary approach. (In the indirect evolutionary approach, which was developed by Güth and Yaari^8^ and Güth^9^, preferences are treated as endogenous in an evolutionary process, while actions are still determined by Nash equilibrium). A divide-a-dollar game, which is also known as a Nash demand game^10^ is one of the most widely used bargaining games, describing a procedure of how to split a dollar. Unlike the ultimatum game that is sequential in the sense that one player proposes a share and then the other player decides whether to accept or reject it, a divide-a-dollar game is simultaneous. The game goes as follows. Player 1 and player 2 simultaneously bid the amount of *x* and *y* respectively to divide a dollar. If the bids turn out to be a feasible division in the sense that $x+y\le 1$, each of them gets the share of their own bid, but otherwise neither gets anything. In this game, both players have a chance to bid, unlike the ultimatum game.

In real situations, bargaining is usually proceeded sequentially and both players have a chance to bid. Therefore, we consider a combination of the two bargaining games in which the two players offer bids sequentially. Unlike the ultimatum game, however, the value of the pie is uncertain. We call this a divide-a-lottery game. So, in a divide-a-lottery game, players bargain for a lottery by bidding sequentially. (Wang et al.^11^ also introduce the randomness associated with the size of pies into the model, but they consider a variant of an ultimatum game, not a variant of a divide-a-dollar game; hence, no sequential bidding in their model).

If two rational players bid sequentially, there is a first mover advantage. So, the first bidder bids $\frac{1}{2}$ and the second bidder bids $\frac{1}{4}$ when the value of the lottery is uniformly distributed on [0, 1]. It results in a high probability of disagreement (i.e., infeasible allocation) due to severe bidding competition. However, a fair player who feels disutility from disparate bargaining shares makes the other player reduce the bid, increasing the probability of agreement. The upshot is that fairness has the role of lowering the bid thereby increasing the expected payoff. As a result, fairness strictly dominates rationality. This is the mechanism through which fairness evolves. However, this result is not robust against even a slight uncertainty about the opponent’s type. Surprisingly, we show a contrasted simulation result that only rational players who are strictly dominated by fair players survive evolutionarily for most of the parameter values if players have even a slight chance of not knowing the opponent’s type. Also, through simulations, we show that moderate proportions of both types coexist evolutionarily over time, and that the population average fitness of this polymorphic population is higher than monomorphic population consisting only of fair types or rational types.

Many authors have demonstrated that fairness evolves in the ultimatum game, not in the divide-a-dollar game nor in the divide-a-lottery game. Nowak et al.^12^ highlighted the role of reputation in the evolution of fairness. If players interact repeatedly, accepting low offers as rational players do can induce the next proposer to make a low offer, so a fair strategy offering and demanding a high share can fare better than a rational strategy. Rand et al.^13^ introduced the possibility of making mistakes to explain the evolution of fairness. Ichinose and Sayama^14^ considered a game which they call not quite ultimatum game in a spatial interaction. Bethwaite and Tompkinson^15^ considered players who are concerned about equity of the allocation similar to our model but did not investigate the evolutionary process of fairness.

Our result that fairness can survive evolutionarily in a more general bargaining situation than in the ultimatum game is a novel finding that does not rely on modeling artifacts in the sense that it is not due to repeated interaction (reputation effect) nor spatial structure of interaction.

## Methods

### Model

We consider a population consisting of a continuum of players of finite measure. Players are classified into two types: rational players (*R*) and fair players (*F*). At any time, they are pairwise matched and play a divide-a-lottery game. The value of a lottery, denoted by *v*, is uncertain. We assume that it is uniformly distributed on [0, 1]. The divide-a-lottery game goes as follows. First, one player (player 1) of the matched pair bids *x* and then the other player (player 2) bids *y*. After that, the value of *v* is realized. If it turns out that $x+y\le v$, they get *x* and *y* respectively and if $x+y>v$, neither gets anything. We assume that each player of the matched pair can be either the first player or the second player with equal probability. Also, we assume symmetric role assignment to make our analysis isolated from role assignment. For (reputation-based) role assignment in the dictator game, see Yang et al.^16^ and Li et al.^17^.

We assume that when a pair is matched, the preference types of the players are known to each other. Since there are two possible types of players, it implies that four pairing combinations are feasible in a stage game.

Let the material payoff of player *i* be $\pi _i (x, y)$ for $i=1,2$. We assume that each player’s material payoff is defined as

1 $$\begin{aligned} \pi _1 (x,y)= x (1-x-y), \end{aligned}$$

2 $$\begin{aligned} \pi _2 (x,y)= y (1-x-y). \end{aligned}$$

This is the expected value of player *i*’s share, since $1-x-y =\mathbb {P}(x+y\le v)$, which is the probability of agreement, i.e., the probability that the allocation by bids is feasible. Although the payoff functions in the divide-a-lottery game are similar to those in the duopoly game, we do not believe that our results will be straightforwardly applied to behavior of firms. In fact, to the best of our knowledge, there is no empirical finding that most firms behave fairly in the duopoly game without maximizing their profits. For endogenous sequencing in the duopoly game, see Dowrick^18^, Boyer and Moreaux^19^, and Hamilton and Slutsky^20^.

When players choose their bids in a stage game, they maximize their subjective utility, not the material payoff. Let $U_{i}^{F}$ represent the subjective utility of fair player *i*. It is defined by

3 $$\begin{aligned} U_{i}^{F}(x,y) = \pi _i (x,y)-\alpha d(x,y), \end{aligned}$$

where $\alpha \ge 0$ is a parameter to represent how much this individual cares about fairness and *d*(*x*, *y*) is a difference between the shares of the two players. (Some authors assume that the disutility is asymmetric, i.e., disutilities when $x > y$ and $x < y$ are different. However, this preference is not really fair). As $\alpha \rightarrow \infty$, the player is fairer. (Conceptually, it is possible that $\alpha =\infty$, but we will restrict our attention to $\alpha \in [0,1]$ to avoid the case that a fair player’s payoff is $-\infty$.) If $\alpha \rightarrow 0$, the player is almost rational. Throughout the article, we assume a simple functional form of $d(x,y)=(x-y)^{2}$.

Let $U_{i}^{R}$ be the subjective utility of a rational player. We assume that the subjective utility of a rational player is the same as his material payoff:

4 $$\begin{aligned} U_{i}^{R}(x, y) = \pi _i (x, y). \end{aligned}$$

## Results

In this section, we analyze the two cases, the case in which each player is informed of the type of the opponent he is facing against (complete information case) and the other case in which each player is not informed of the opponent’s type (incomplete information case).

### Complete information about the opponent’s type

We consider two symmetric matching cases, rational player vs. rational player and fair player vs. fair player, and one asymmetric matching case, rational player vs. fair player.

#### Rational player vs. rational player

Since a stage game is sequential, we use backward induction to obtain the subgame perfect equilibrium. If rational player 1 plays against rational player 2, given the bid of player 1, *x*, player 2 seeks to maximize his material payoff by choosing

5 $$\begin{aligned} y^R (x) = \arg \max _y \pi _2 (x, y)=y(1-x-y), \end{aligned}$$

so we obtain player 2’s best response as a function of *x*:

6 $$\begin{aligned} y^{R}(x)=\frac{1-x}{2}. \end{aligned}$$

Taking account of this response, player 1 chooses *x* to maximize

7 $$\begin{aligned} \pi _1 (x, y^{R}(x))=x(1-x-y^{R}(x))=x\left[ 1-x-\frac{1-x}{2}\right] . \end{aligned}$$

Therefore, equilibrium bids are $x^* =\frac{1}{2}$ and $y^* =y^R (x^* )=\frac{1}{4}$.

Let $\pi ^{RR}$ be the material expected payoff of a rational player playing against another rational player. The material payoffs of rational player 1 and player 2 are $\pi _1 = \frac{1}{8}$ and $\pi _2 = \frac{1}{16}$ respectively. This shows the first mover advantage. The first mover can choose a higher bid than the second mover who is passive. By increasing his bid before player 2, he can enjoy a strategic advantage. Since a player can be player 1 or player 2 with equal probability, their expected value of the material payoff is $\pi ^{RR}=\frac{1}{2}\left( \pi _{1}^{RR} +\pi _{2}^{RR} \right) = \frac{1}{2}\left( \frac{1}{8}+\frac{1}{16}\right) =\frac{3}{32}$.

#### Fair player vs. fair player

If a fair player plays against another fair player, player 2 chooses his bid to maximize his subjective utility, taking his opponent’s bid *x* as given:

8 $$\begin{aligned} U_{2}^{FF}(x, y)=y(1-x-y)-\alpha (x-y)^{2}. \end{aligned}$$

From the first-order condition for maximizing (8), we obtain fair player 2’s best response function as

9 $$\begin{aligned} y^{F}(x)=\frac{1+(2\alpha -1)x}{2(1+\alpha )}. \end{aligned}$$

Taking this response of player 2 into account, player 1 chooses *x* to maximize

10 $$\begin{aligned} U_{1}^{FF}(x, y^{F}(x))&= x(1-x-y^{F}(x))-\alpha (x-y^F (x))^{2}\nonumber \\&= x\left[ 1-x-\frac{1+(2\alpha -1)x}{2(1+\alpha )}\right] -\alpha \left[ x- \frac{1+(2\alpha -1)x}{2(1+\alpha )}\right] ^{2}. \end{aligned}$$

Therefore, we obtain equilibrium bids as $x^* (\alpha )=\frac{2\alpha ^{2}+6\alpha +1}{8\alpha ^{2}+19\alpha +2}$ and $y^* (\alpha )=y^F (x^* (\alpha ))=\frac{4\alpha ^{2}+14\alpha +1}{2(8\alpha ^{2}+19\alpha +2)}$ where $\frac{\partial x^{*}(\alpha )}{\partial \alpha }<0$, $x^* (0)=\frac{1}{2}$ and $x^* (1)= \frac{9}{29}<\frac{1}{3}$ and $y^* (1)=\frac{19}{58}>\frac{9}{29}$. As $\alpha$ gets larger, i.e., players get fairer, player 1 bids less to reduce the first mover advantage, and if $\alpha = 1$, the first mover advantage disappears completely, because $x^* (1)<y^* (1)$. (It is easy to check that $y^* (\alpha )$ is not monotonic with respect to $\alpha$).

Let $\pi ^{FF}$ be their expected value of the material payoff. Then, we can compute $\pi ^{FF}$ as $\pi ^{FF}=\frac{1}{2}(\pi _{1}^{FF}+\pi _{2}^{FF})= \frac{(8\alpha ^{2}+26\alpha +3)(8\alpha ^{2}+12\alpha +1) }{8(8\alpha ^{2}+19\alpha +2)^{2}}$.

#### Rational player vs. fair player

If player 1 is rational and player 2 is fair, we know from (9) that $y^{F}(x)=\frac{1+(2\alpha -1)x}{2(1+\alpha )}$. Calculating this response of player 2, player 1 chooses *x* to maximize

11 $$\begin{aligned} U^{RF}(x, y^{F}(x))=x(1-x-y^{F}(x))=x\left[ 1-x-\frac{1+(2\alpha -1)x}{2(1+\alpha )}\right] . \end{aligned}$$

Therefore, we obtain $x^* (\alpha )=\frac{2\alpha +1}{2(4\alpha +1)}$ and $y^* (\alpha )= y^{F}(x^* (\alpha ))= \frac{4\alpha ^{2}+8\alpha +1}{4(\alpha +1)(4\alpha +1)}$. Note that $\lim _{\alpha \rightarrow 0} x^* (\alpha )=\frac{1}{2}$, $\lim _{\alpha \rightarrow 0} y^* (\alpha )=\frac{1}{4}$, $\lim _{\alpha \rightarrow 1} x^* (\alpha )=\frac{3}{10}$, and $\lim _{\alpha \rightarrow 1} y^* (\alpha )=\frac{13}{40}>\frac{3}{10}$. (It is interesting to note that $x^* (\frac{1}{2})=y^* (\frac{1}{2})= \frac{1}{3}$, which is identical to the equilibrium outcome of the simultaneous divide-a-lottery game). Again, the first mover advantage disappears in the case of fair player 2. Also, we obtain $\pi _{1}^{RF}=\frac{(2\alpha +1)^{2}}{8(4\alpha +1)(\alpha +1)}$ and $\pi _{2}^{RF} =\frac{(4\alpha ^{2}+8\alpha +1)(2\alpha +1)}{16(\alpha +1)^{2}(4\alpha +1)}$.

On the other hand, if player 1 is fair and player 2 is rational, equilibrium bids are $x^* (\alpha ) =\frac{1+3\alpha }{2+9\alpha }$ and $y^* (\alpha )= \frac{1+6\alpha }{2(2+9\alpha )}$, and accordingly the equilibrium material payoffs are $\pi _{1}^{FR}=\frac{(3\alpha +1)(6\alpha +1)}{2(9\alpha +2)^{2}}$ and $\pi _{2}^{FR} = \frac{(6\alpha +1)^{2}}{4(9\alpha +2)^{2}}$.

Let $\pi _{R}$ and $\pi _{F}$ be the equilibrium expected material payoffs of a rational player and a fair player respectively when a rational player and a fair player are matched. Then, they can be computed as

12 $$\begin{aligned} \pi _{R}= \frac{1}{2}\left( \pi _{1}^{RF}+\pi _{2}^{FR}\right) , \end{aligned}$$

13 $$\begin{aligned} \pi _{F}= \frac{1}{2}\left( \pi _{1}^{FR}+\pi _{2}^{RF}\right) . \end{aligned}$$

Table 1 shows the computation results of material payoffs.

**Figure 1:**
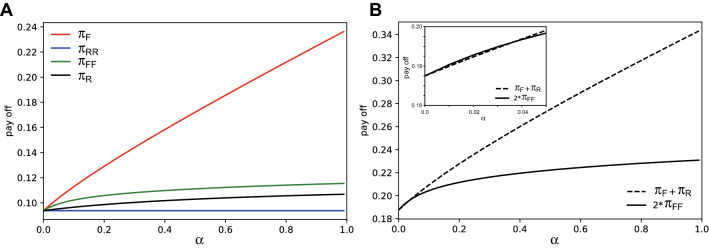
Values of material payoffs for complete information cases. For the comparison of material payoffs, we observe the values of each material payoff (**A**), and ($\pi _F + \pi _R$)-vs. $2*\pi _{FF}$ (**B**, inset), according to the parameter $\alpha$.

If the fitness of a player is determined by his material payoff, we can see from Table 1 that fairness is the dominant strategy for any $\alpha \in [0,1]$. For the numerical proof, Fig. 1A shows that $\pi _{F} >\pi _{RR}$ and $\pi _{FF}>\pi _{R}$ for any $\alpha \in (0,1]$. So, if strategies evolve in proportion to the material payoffs, only fairness can survive evolutionarily in the long run. Here, as a dynamic solution concept, we are using a long-run asymptotic (local) attractor that can be roughly defined by the population distribution to which an initial distribution converges over time whenever it starts from the neighborhood.

**Proposition 1 **Only fair players can survive evolutionarily if a randomly matched pair in a population plays a divide-a-lottery game and the players know each other’s type.

The analytic proof is omitted, because it is well known that a strict Nash equilibrium is an evolutionarily stable strategy (ESS) by Maynard Smith and Price^21^ which is a long-run asymptotic attractor. In this game, (*F*, *F*) is a strict Nash equilibrium.

At this moment, it is worthwhile to compare this game with the prisoners’ dilemma (PD) game. In a PD game, cooperation (*C*) is strictly dominated by defection (*D*), but (*C*, *C*) yields higher fitness than (*D*, *D*). In other words, (*C*, *C*) is the collectively rational outcome (socially efficient outcome), whereas (*D*, *D*) is the individually rational outcome (privately optimal outcome). The discrepancy is where social dilemma comes from. In our divide-a-lottery game, fairness strictly dominates rational behavior, but unlike the PD game, the individually rational outcome (*F*, *F*) yields higher fitness than (*R*, *R*). This is the main difference from the PD game. Also, it is interesting to note that the collectively rational outcome in this game is (*F*, *R*) and (*R*, *F*), not (*F*, *F*), for most parameter values except for very small values of $\alpha \in (0, 0.04)$ (Fig. 1B). This implies that for most values of $\alpha$, a polymorphic population is socially better than a monomorphic population consisting only of fair players in terms of the population average fitness.

Since the complete information assumption that drives this result is too strong to properly capture the real world phenomenon, we relax the assumption and consider the incomplete information case in the next section.

### Incomplete information about the opponent’s type

In this section, we assume that players cannot tell the type of the opponent but only know the proportion of each type. Let *p* be the proportion of fair players in the population. A rational player 1 chooses $x^{R}$ to maximize

14 $$\begin{aligned} E(\pi _1 )&= (1-p)x(1-x-y^R (x))+px(1-x-y^F (x)) \nonumber \\&= (1-p)x\left[ 1-x-\frac{1-x}{2}\right] +px\left[ 1-x-\frac{1+(2\alpha -1)x}{2(1+\alpha )}\right] . \end{aligned}$$

The first order condition leads to

15 $$\begin{aligned} x^R = \frac{\alpha p+\alpha +1}{6\alpha p+2\alpha +2}. \end{aligned}$$

A fair player chooses $x^F$ to maximize

16 $$\begin{aligned} E(\pi _1 )&= (1-p)\left[ x(1-x-y^R (x))-\alpha \left( x-y^R (x)\right) ^{2}\right] +p\left[ x(1-x-y^F (x))-\alpha (x-y^F (x))^{2}\right] \nonumber \\&= (1-p)\left[ x\left( 1-x-\frac{1-x}{2}\right) -\alpha (x-\frac{1-x}{2})^{2}\right] \nonumber \\ & \quad +p\left[ x\left( 1-x-\frac{1+(2\alpha -1)x}{2(1+\alpha )}\right) -\alpha \left( x-\frac{1+(2\alpha -1)x}{2(1+\alpha )}\right) ^{2}\right] . \end{aligned}$$

Thus, we obtain

17 $$\begin{aligned} x^F =\frac{3\alpha ^{3}(1-p)+\alpha ^{2}(7-5p)+\alpha (p+5)+1}{9\alpha ^{3}(1-p)+4\alpha ^{2}(5-3p)+\alpha (6p+13)+2}. \end{aligned}$$

Substituting (15) and (17) into (6) and (9), we get

18 $$\begin{aligned} y^R (x^R )= \frac{5\alpha p+\alpha +1}{4(3\alpha p+\alpha +1)}, \end{aligned}$$

19 $$\begin{aligned} y^R (x^F )= \frac{6\alpha ^{3}(1-p)+\alpha ^{2}(13-7p)+\alpha (8+5p)+1}{2[9\alpha ^{3}(1-p)+4\alpha ^{2}(5-3p)+\alpha (6p+13)+2]},\end{aligned}$$

20 $$\begin{aligned} y^F (x^R )= \frac{2\alpha ^{2}p+2\alpha ^{2}+5\alpha p+3\alpha +1}{4(\alpha +1)(3\alpha p+\alpha +1)},\end{aligned}$$

21 $$\begin{aligned} y^F (x^F )= \frac{6\alpha ^{4}(1-p)+4\alpha ^{3}(5-4p)+\alpha ^{2}(23-5p)+5\alpha (p+2)+1}{2(\alpha +1)[9\alpha ^{3}(1-p)+4\alpha ^{2}(5-3p)+\alpha (6p+13)+2]}. \end{aligned}$$

Let $\pi _{i}^{IR}$ and $\pi _{i}^{IF}$ be the equilibrium material payoffs of a rational (or fair respectively) player *i* where $i=1,2$ in the case of incomplete information. Then, we can compute the expected value of the material payoff of each type as

22 $$\begin{aligned} \pi ^{IR}= \frac{1}{2}(\pi _{1}^{IR}+\pi _{2}^{IR}), \end{aligned}$$

23 $$\begin{aligned} \pi ^{IF}= \frac{1}{2}(\pi _{1}^{IF}+\pi _{2}^{IF}), \end{aligned}$$

where

24 $$\begin{aligned} \pi _{1}^{IR}= (1-p)x^R (1-x^R -y^R (x^R ))+px^R (1-x^R -y^F (x^R )), \end{aligned}$$

25 $$\begin{aligned} \pi _{2}^{IR}= (1-p)y^R (x^R )(1-x^R -y^R (x^R ))+py^R (x^F )(1-x^F -y^R (x^F )),\end{aligned}$$

26 $$\begin{aligned} \pi _{1}^{IF}= (1-p)x^F (1-x^F -y^R (x^F ))+px^F (1-x^F -y^F (x^F )),\end{aligned}$$

27 $$\begin{aligned} \pi _{2}^{IF}= (1-p)y^F (x^R ) (1-x^R -y^F (x^R ))+py^F (x^F ) (1-x^F -y^F (x^F )). \end{aligned}$$

**Figure 2:**
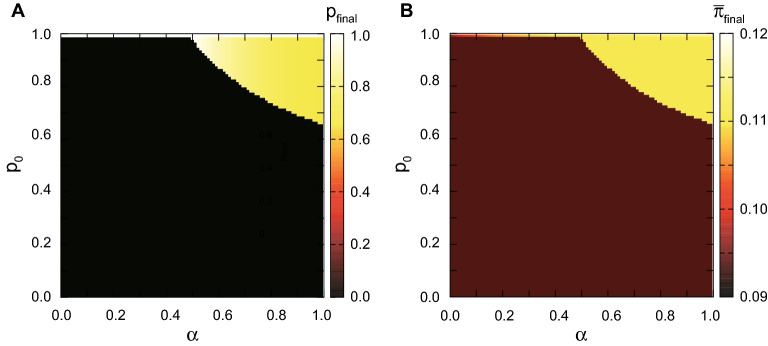
Numerical observation of final fraction of fair players ($p_{final}$) and the average fitness of the population ($\bar{\pi }_{final}$) from the replicator dynamics of incomplete case. (**A**) For each pair of parameter values $p_0$ and $\alpha$, the final fractions of fair players in equilibrium are presented as color scale. The black region means only rational players can survive in final state while both types of players can coexist in the yellow region. (**B**) For each pair of parameter values $p_0$ and $\alpha$, the average fitness of the population ($\bar{\pi }_{final}$) in equlibrium are presented as color scale, too. The yellow region denotes a higher average fitness compared to the brown region. It indicates that evolution does not favor the population distribution with the highest average fitness, when players interact globally.

Let us consider the following replicator dynamics

28 $$\begin{aligned} p_{t+1}=p_t \frac{\pi ^{IF}}{\bar{\pi ^{I}}}, \end{aligned}$$

where $\bar{\pi ^{I}}=(1-p_{t})\pi ^{IR} +p_t \pi ^{IF}$. Then, we can find the limiting distribution of *R* and *F*. Figure 2A shows our simulation results that only the monomorphic population consisting only of rational players emerge as a result of evolution for most parameter values (black region), while a polymorphic population can emerge for high values of $\alpha$ (degree of fairness) and $p_{0}$ (initial proportion of fairness) (yellow region). Figure 2B shows the average of material payoffs in the limiting states for various combinations of $(p_0,\alpha )$. It implies that the polymorphic population consisting of mixture of rational players and fair players is better than the monomorphic population consisting solely of rational players in terms of the population average fitness. This implies that the population distribution is very unlikely to converge to the monomorphic population distribution that yields the highest population average fitness when players interact with each other globally with equal probabilities.

This result is quite puzzling. In this game, fairness strictly dominates rationality in the case of complete information, as shown in Tab.1. It means that it is better for a player to play fairly, regardless of the opponent’s type. This seems to imply that a player does not need to know the opponent’s type, because he will get a better payoff when he plays fairly than when he plays rationally. Then, how can rational players still survive evolutionarily if players cannot be sure of the opponent’s type? Specifically, when $p_{0}\approx 0$, how can it be possible that $\pi ^{IR} >\pi ^{IF}$ in the case of incomplete information, although $p_0 \approx 0$ means that the population consists only of rational players so Table 1 seems to suggest that only fair players can survive because $\pi _F >\frac{3}{32}$? The answer for this puzzle can be found from the difference between $\pi _F =\frac{1}{2}(\pi _{1}^{FR}+\pi _{2}^{RF})$ given in (13) and $\pi ^{IF} = \frac{1}{2}(\pi _{1}^{IF}+\pi _{2}^{IF})$ given in (23) when $p_0 \approx 0$. Note that $\pi _{2}^{RF}=y^F (x^R )(1-x^R -y^F (x^R ))$ and $\pi _{2}^{IF}\approx y^F (x^R )(1-x^R -y^F (x^R ))$ given in (27) when $p_{0}\approx 0$. Although they look the same, the values of $x^R$ in the two formulas are different, depending on what the opponent is. In the former (in the complete information case), it is computed from the assumption that the second mover is fair. (We used the notation $x^* (\alpha )$ instead of $x^R$ in the analysis of complete information case to distinguish them). In the latter (in the incomplete information case), however, the rational player chooses $x^{R}$, expecting that the second mover is highly likely to be rational when $p_0 \approx 0$. (In other words, the true opponent type and the expected opponent type can be different in the case of incomplete information, whereas it is not possible in the case of complete information). So, $x^R$ in this case is larger and thus it is more likely to be rejected. Hence, $\pi _{2}^{RF}$ is lower, so is the fitness of a fair player in the case of incomplete information.

The overall intuition for the case of incomplete information goes as follows. If players have complete information about the opponent’s type, a rational player (player 1) bids very high to the rational opponent, so rational player 2 is severely exploited, while he bids lower to the fair opponent, because he knows that the fair opponent (fair player 2) will bid so high that his high bid would be very likely to lead to a failure in bargaining. However, if players have incomplete information about the opponent’s type, a rational player who is unsure of the opponent’s type must bid lower than when he faces a rational opponent, and so a rational player 2 is not very much exploited. This is one of the main reasons why rational players fare better under incomplete information. Similarly, a fair player who is unsure of the opponent’s type can bid higher if there is a high probability that the opponent is a rational type. So, incomplete information can have the role of making a rational player play more like a fair player and making a fair player play more like a rational player. (If a player is the second mover, his decision does not depend on the opponent’s type. So, the incomplete information of the opponent’s type does not affect his choice under complete information).

Under complete information, a rational player earns a low material payoff because he is significantly exploited when he is the second mover. On the other hand, under incomplete information, he is not so much exploited because the opponent is not sure whether he is rational or not. However, if the proportion of fair players is very high, it becomes an almost complete information game, and the advantage of fairness in the case of complete information is almost balanced with the advantage of rationality in the case of incomplete information, so both of the two types can survive and evolve over time.

**Table 1:** Payoff matrix (material payoffs).

	*R*	*F*
*R*	$\frac{3}{32}, \frac{3}{32}$	$\pi _{R}, \pi _{F}$
*F*	$\pi _{F}, \pi _{R}$	$\pi ^{FF}, \pi ^{FF}$

$$\pi _R=\frac{612\alpha ^{4}+924\alpha ^{3}+441\alpha ^{2}+86\alpha +6}{16(\alpha +1)(4\alpha +1)(9\alpha +2)^{2}}$$, $$\pi _F =\frac{1224\alpha ^{5}+3492\alpha ^{4}+3106\alpha ^{3}+1169\alpha ^{2}+196\alpha +12}{32(\alpha +1)^{2}(4\alpha +1)(9\alpha +2)^{2}}$$, $$\pi ^{FF}= \frac{(8\alpha ^{2}+26\alpha +3)(8\alpha ^{2}+12\alpha +1) }{8(8\alpha ^{2}+19\alpha +2)^{2}}$$

### Local interaction on a network

**Figure 3:**
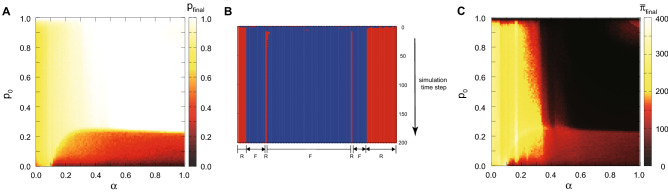
Results of local interaction of a network. (**A**) Simulation result of final fraction of fair players ($p_{final}$) according to the parameter $\alpha$ and $p_0$ for the given interaction radius $r_{inter}=10$ and information radius $r_{infor}=1$. The white region indicates that only fair players can survive while both types of players can coexist in the yellow and orange region. (**B**) For the given $\alpha =0.1$ and $p_0 = 0.1$, we illustrate how individual players change their types according to simulation steps. The blue (red) circle denotes fair (rational) players respectively. (**C**) Simulation result of average final fitness of the population ($\bar{\pi }_{final}$) according to the parameter $\alpha$ and $p_0$ for the given interaction radius $r_{inter}=10$ and information radius $r_{infor}=1$. The yellow region indicates that the population average fitness is very high compared to the other regions shown in red and black.

In this section, we consider a simple network structure on which players interact locally to play a divide-a-lottery game. For recent studies on local interaction in other situations such as the prisoners’ dilemma game with social diversity or the snowdrift game, see Perc and Szolnoki^22^ and Hauert and Doebeli^23^. Initially, there are $n (=100)$ players on a circle. The type of each player (*R* or *F*) is assigned randomly according to the pre-assigned ratio of fair players ($p_{0}$). Here, we introduce two parameters, interaction radius ($r_{inter}$) and information radius ($r_{infor}$). Each player interact only with neighbors within the given $r_{inter}$. As we investigated the cases of complete and incomplete information about the opponent’s type in previous subsections, players know the type of her neighbor within the given $r_{infor}$ and does not know outside of the length $r_{infor}$. Then, players observe their own fitness and their neighbors’ within the interaction radius after they play the divide-a-lottery game with the neighbors. Finally, each player decides to change her type by imitating the type of her neighbors when the average fitness of her neighbors of different types is greater than the fitness of her own type. The simulation continues until the dynamics becomes stable. We observe the fraction of fair players at the final step of simulation ($p_{final}$) with varying two parameters of initial fraction of fair players ($p_{0}$) and fairness careness $\alpha$ as the average of $10^3$ times of ensembles (see Fig. 3).

For given $r_{inter}=10$ and $r_{infor}=1$, we can find that there are mainly two phases of final states: (1) At higher $\alpha$ and higher $p_{0}$, only fair players survive (white region) and (2) rational players are predominant with higher $\alpha$ and lower $p_{0}$ values. This means that the emergence of fairness can be determined by the given condition of $\alpha$ and $p_{0}$. Note that if $\alpha$ is too high, it may not be good for a fair player because his bid becomes lower (Fig. 3A). For different combinations of $r_{inter}$ and $r_{infor}$, the results are qualitatively similar (see Fig. S1). At lower values of $\alpha$, both types of players can coexist, as depicted in yellow color in Fig. 3A. This is also confirmed in Fig. 3B which illustrates how some initial distribution reaches a stationary spatial distribution over time by simulations. Note that in the case of some nodes, they oscillate unstably at first, and then become stable and maintain their types over time. Also, Fig. 3C shows that a polymorphic population consisting of both of rational players and fair players is better than the monomorphic population consisting only of fair players in terms of population average fitness. This is mainly because $\pi _F + \pi _R > 2\pi _{FF}$ for most parameter values of $\alpha$. This figure implies that the evolution process in a local interaction favors the ultimate population distribution that yields high population average fitness.

**Figure 4:**
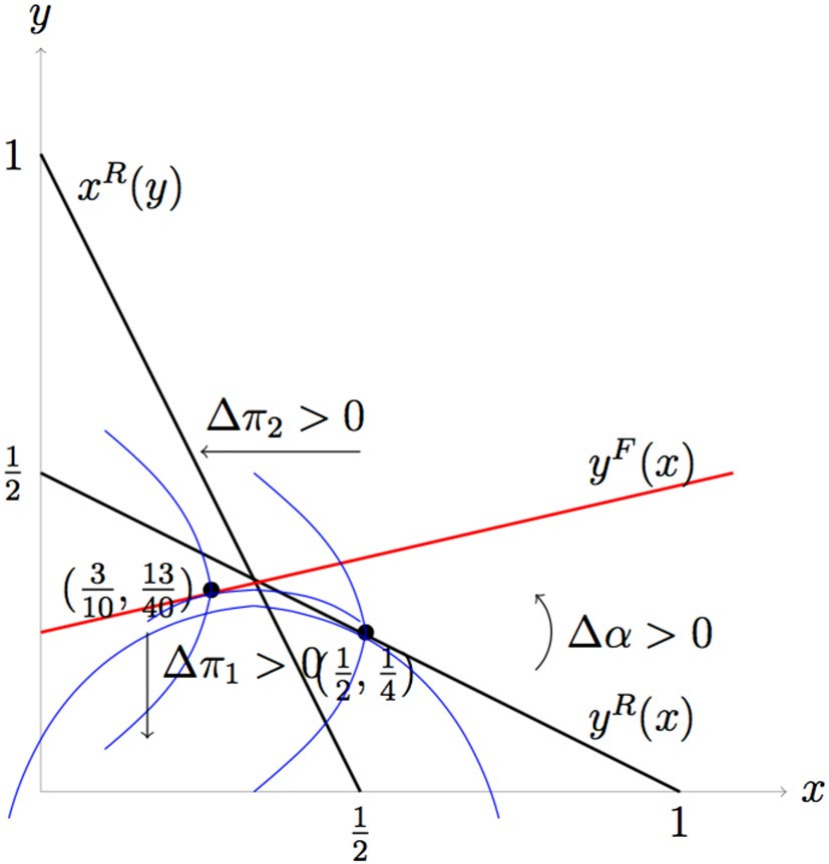
Comparison of bids and payoffs with rational opponent and fair opponent. Black lines denoted by $x^{R}(y)$ and $y^{R}(x)$ are reaction curves when both players are rational, and the red line denoted by $y^{F}(x)$ is the reaction curve when the player is fair. Blue curves are the two players’ indifference curves that yield same utility to each player. The cap-shaped curve is player 1’s indifference curve. His utility increases as it moves downward towards *x*-axis, while player 2’s utility increases as it moves inward towards *y*-axis. If the second player is rational, his reaction curve is $y^{R}(x)$, so player 1 chooses the point that gives the maximum utility on $y^{R}(x)$. It is the tangent point of $y^{R}(x)$ and the indifference curve, (1/2, 1/4) if $\alpha =0$. If the second player is fair, the tangent point of $y^{F}(x)$ and the indifference curve is (3/10, 13/40) if $\alpha =1$.

Before closing this section, we highlight the intuition for why fair players can survive evolutionarily. If two rational players play the game, there is a first mover advantage. Player 1 preempts an advantageous position by making a high bid which makes player 2 makes a low bid. However, if player 2 is fair, player 1 cannot bid high because he knows that the fair opponent will not reduce his bid very much due to his concerns for fairness. This makes player 1 reduce his bid. So, as $\alpha$ is larger, i.e., player 2 is fairer, player 1 reduces his bid more so that player 2 increases his bid, and thus player 1’s payoff gets smaller and player 2’s payoff gets larger, until they bid the same when $\alpha =\frac{1}{2}$. If $\alpha$ exceeds $\frac{1}{2}$, player 2 begins to reduce his bid, although he still bids more than player 1. So, player 1’s payoff begins increasing as $\alpha$ gets larger. Figure 4 shows this intuition.

**Figure 5:**
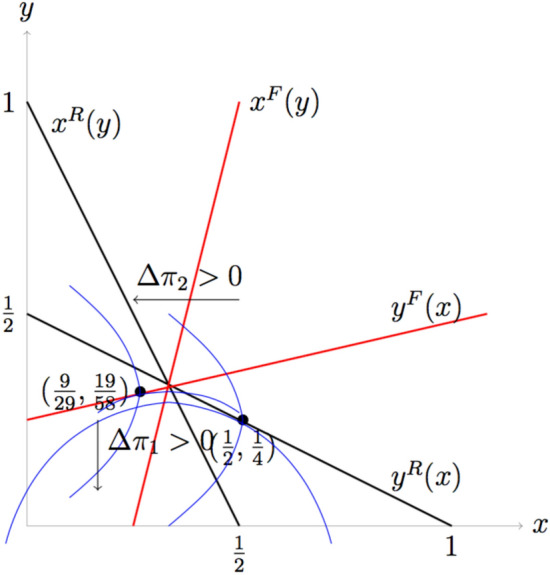
Equilibrium bids when both players are fair If player 1 is fair, his indifference curve has the zero slope on $x^{F}(y)$, not on $x^{R}(y)$, because $x^{F}(y)$ is the optimal point for him given *y*. This figure shows the equilibrium bids (9/29, 19/58), which is the tangent point of player 1’s indifference curve and player 2’s reaction curve $y^{F}(x)$, when both are fair, if $\alpha =1$.

If both players are fair, the situation is similar. In this case, the best response curves are both upward sloping. Since player 1 takes the best response function of player 2 as given, he will choose the optimal point along the best response curve of player 2 which is lower than $x=\frac{1}{3}$, the intersection of the two best response curves. Since the best response curve of player 1 is upward sloping, low *x* means low *y*. Since both *x* and *y* are reduced, both fair players get higher payoffs than rational players. It is illustrated in Fig. 5.

## Discussion

In this paper, we demonstrated that fair players can survive evolutionarily in a divide-a-lottery game. Moreover, we showed that rational players can also survive in the environment in which the bargaining players do not know each other’s type until they play the bargaining game with the opponent, depending on the initial population distribution. Considering the reality that players often compete with their local neighbor whose type is not known (until they interact) and for the pie the value of which is uncertain, we believe that this result gives a sensible prediction in the real world.

## Supplementary Information

Supplementary Figure S1.

## Data Availability

The datasets used and/or analysed during the current study available from the corresponding author on reasonable request.
