# Supplementary material for: Evolution of fairness in the divide-a-lottery game
Source: Sci Rep. 2023 Apr 29;13:7048. doi: 10.1038/s41598-023-34131-w (PMC10148846; doi:10.1038/s41598-023-34131-w)
Supplement: Supplementary file 1 — Supplementary Figure S1. [file 41598_2023_34131_MOESM1_ESM.pdf]

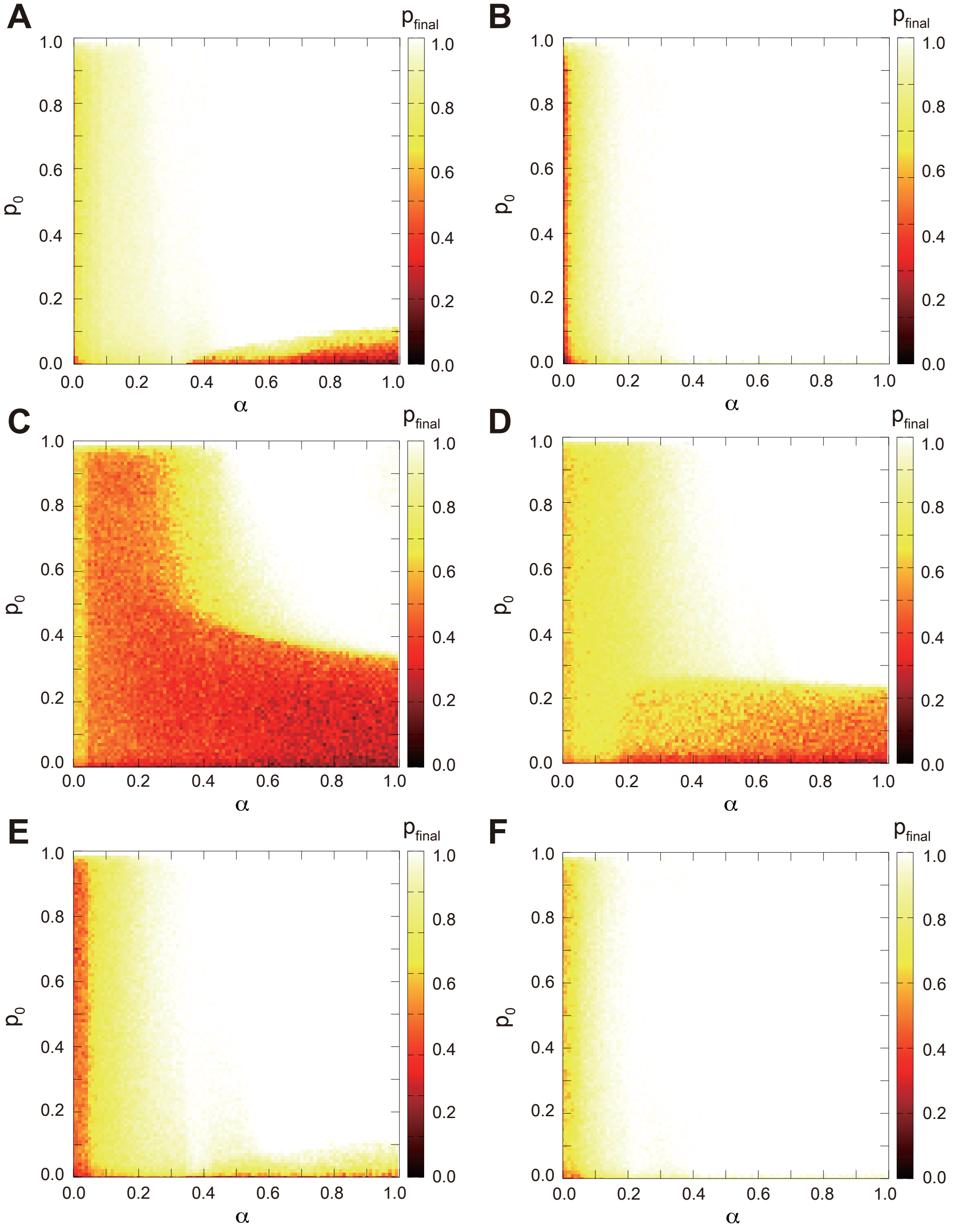

Figure S1: Simulation result of final fraction of fair players (p_{final}) according to the parameter α and p_0 for the given interaction and information radius, $r_{inter} = 10$, $r_{infor} = 2$ (A), $r_{inter} = 10$, $r_{infor} = 4$ (B), $r_{inter} = 20$, $r_{infor} = 1$ (C), $r_{inter} = 20$, $r_{infor} = 2$ (D), $r_{inter} = 20$, $r_{infor} = 4$ (E), and $r_{inter} = 20$, $r_{infor} = 8$ (F).
